# Minor Role of Plasminogen in Complement Activation on Cell Surfaces

**DOI:** 10.1371/journal.pone.0143707

**Published:** 2015-12-04

**Authors:** Satu Hyvärinen, T. Sakari Jokiranta

**Affiliations:** Department of Bacteriology and Immunology, and Research Programs Unit, Immunobiology, University of Helsinki, Helsinki, Finland; National Cerebral and Cardiovascular Center, JAPAN

## Abstract

Atypical hemolytic uremic syndrome (aHUS) is a rare, but severe thrombotic microangiopathy. In roughly two thirds of the patients, mutations in complement genes lead to uncontrolled activation of the complement system against self cells. Recently, aHUS patients were described with deficiency of the fibrinolytic protein plasminogen. This zymogen and its protease form plasmin have both been shown to interact with complement proteins in the fluid phase. In this work we studied the potential of plasminogen to restrict complement propagation. In hemolytic assays, plasminogen inhibited complement activation, but only when it had been exogenously activated to plasmin and when it was used at disproportionately high concentrations compared to serum. Addition of only the zymogen plasminogen into serum did not hinder complement-mediated lysis of erythrocytes. Plasminogen could not restrict deposition of complement activation products on endothelial cells either, as was shown with flow cytometry. With platelets, a very weak inhibitory effect on deposition of C3 fragments was observed, but it was considered too weak to be significant for disease pathogenesis. Thus it was concluded that plasminogen is not an important regulator of complement on self cells. Instead, addition of plasminogen was shown to clearly hinder platelet aggregation in serum. This was attributed to plasmin causing disintegration of formed platelet aggregates. We propose that reduced proteolytic activity of plasmin on structures of growing thrombi, rather than on complement activation fragments, explains the association of plasminogen deficiency with aHUS. This adds to the emerging view that factors unrelated to the complement system can also be central to aHUS pathogenesis and suggests that future research on the mechanism of the disease should expand beyond complement dysregulation.

## Introduction

Plasminogen is a 92-kDa glycoprotein that circulates the vasculature at a concentration of 0.2 mg/ml (2 μM). The closed structure enables plasminogen to maintain its zymogenic form, but removal of an N-terminal peptide exposes the cleavable Arg561-Val562 bond and allows some plasmin activation by tissue- or urokinase-type plasminogen activators (tPA and uPA) [1]. Efficient formation of plasmin, however, requires that plasminogen and tPA form a ternary complex with fibrin. Plasmin can also be produced on endothelial cells on which Annexin II—S100A10 heterotetramers co-localize plasminogen with tPA or receptor-bound uPA [2]. By cleaving fibrin, plasmin enhances its own activation as the exposed C-terminal lysines function as binding sites for new plasminogen and tPA molecules [3]. The thrombin-activatable fibrinolysis inhibitor (TAFI) counteracts this effect by removing lysines. The primary physiological inhibitor of plasmin in plasma is α2-antiplasmin (α2-AP; 1 μM), that forms stable inactive complexes with free plasminogen [4]. α2-AP can also be covalently linked to fibrin to modulate fibrinolysis [5]. Plasminogen activator inhibitor 1 (PAI-1) exerts its effect indirectly by inactivating the activators of plasminogen.

Plasmin activity has been implicated in, for example, tissue remodeling, wound healing, angiogenesis, and cancer invasion [6] but also in cell injury. This can probably be attributed to the ability of plasmin to degrade components of the extracellular matrix and basal membrane, and to activate matrix metalloproteases. Plasmin is probably best known for its critical role in fibrinolysis. With its ability to digest fibrin fibers, plasmin controls the extent of coagulation and is of central importance for solubilization of blood clots. Therefore it is somewhat surprising that plasminogen deficiency as a sole abnormality is mainly related to ligneous conjunctivitis [7], but does not seem to increase risk of thrombosis in human subjects [8]. Interestingly, plasminogen deficiency was recently associated with the disease atypical hemolytic uremic syndrome (aHUS) [9].

aHUS is a rare systemic disease characterized by hemolysis, thrombocytopenia and renal impairment [10]. Central to aHUS is dysregulation of the complement system on host cells [11]. Complement is a network of over 30 serum proteins that normally act, for example, in protecting the body against invading microbes and in removal of damaged cells (reviewed by Ricklin et al. [12]). Different arms of the system are activated on various targets, leading to marking these surfaces with the opsonin C3b and assembly of lytic pores, i.e. C5b-9 complexes, into cell membranes. To prevent harmful complement attack against normal self cells, its activation is tightly controlled by multiple soluble and membrane-bound regulators. With the disease aHUS, roughly 70% of patients have one or several abnormalities of complement genes [13]: loss-of-function mutations in regulators and gain-of-function mutations in activators lead to uncontrolled activation of the complement cascade [10].

Considering the thrombotic nature of aHUS, it is not surprising that, besides complement factors, an increasing number of proteins of the coagulative and fibrinolytic systems are being linked to the disease. The most recent example is plasminogen with four aHUS-patients showing variation in the respective protein encoding gene [9]. Three of the plasminogen variants reported were known from earlier literature to cause plasminogen deficiency, and the authors suggested that in these aHUS-cases degradation of thrombi was compromised due to reduced fibrinolytic activity. Support for impaired fibrinolysis to be involved in aHUS was obtained in a study where the ratio of antifibrinolytic PAI-1 to profibrinolytic tPA was shown to be markedly increased in patient biopsies at mRNA-level [14]. Data on fibrinolysis in typical HUS, on the other hand, is controversial [9]. In some aHUS-patients, a prothrombotic state of endothelial cells and platelets due to mutations in the gene encoding diacylglycerol kinase epsilon (DGKε) seems to cause the disease [15]. Further, the coagulation factor XII and the anticoagulant glycoprotein thrombomodulin have been associated with aHUS [9, 16, 17].

Versatile crosstalk has been proposed to exist between the coagulation and complement systems [18, 19]. Of the aHUS-associated coagulation pathway proteins, factor XII, for example, activates C1 [20] and thrombomodulin assists in inhibition of C3b, C3a, and C5a [16]. Weak enhancement of cleavage of purified C3b in the fluid phase has been reported for the newly aHUS-linked protein plasminogen as well [21]. As for plasmin, this powerful protease cleaves both intact and activated forms of multiple complement proteins [21–28]. Streptokinase activation of plasminogen to plasmin has, for example, been shown to reduce the activities of the central complement members C1, C2, C3 and C4 [22]. The physiological relevance of the interactions of plasmin with complement has been experimented mainly in bacterial settings, with several studies showing plasmin-mediated reduction in C3b-deposition [29–31]. Plasmin has also been reported to inhibit complement mediated erythrocyte lysis in highly diluted human serum [21]. In all these assays, however, plasmin was artificially activated. Multiple bacteria possess specific proteins with which the plasminogen acquired on their surfaces could be activated to plasmin, at least during some phase of infection [32]. Erythrocytes, on the other hand, do not practically bind plasminogen [33]. These earlier results led us to ask whether plasminogen could regulate complement activation on surfaces of human cells capable of both binding and activating this zymogen.

Of the three types of cells damaged in aHUS, abnormal complement activation is involved in injury to endothelial cells especially lining the glomerular microvasculature [10] and to platelets [34]. Hemolysis in aHUS-patients, on the other hand, is generally referred to be mechanical in nature [13], although a sensitizing cell stiffening role for C3b-deposition in erythrocyte rupture cannot be ruled out [35]. Fibrin, an important constituent of thrombi, has also been claimed to activate the complement system [36, 37], although it has not been considered a target in aHUS. In this work, we set out to experiment the potential of plasminogen to regulate complement activation on the surfaces relevant to aHUS. Our results show that, in conditions resembling those found physiologically, plasminogen does not restrict propagation of the complement cascade on erythrocytes or endothelial cells but has a very modest inhibitory effect on complement activation on platelets.

## Materials and Methods

### Ethics Statement

The appropriate Institutional Review Board of the University of Helsinki had approved the extraction of and the studies with the human umbilical vein endothelial cells (HUVECs), and the protocols using blood had been approved by the Section for Research of the Helsinki University Central Hospital Laboratory (project TYH7214). A written informed consent was obtained from all donors in accordance to the Declaration of Helsinki.

### Materials

Plasminogen, plasmin, human HMW urokinase, and human tissue plasminogen activator (tPA) were from Molecular Innovations. Plasminogen and plasmin were also purified / prepared from human plasma (see below). Human fibrinogen and alpha thrombin were from Enzyme Research. S-2251 was from Chromogenix. BSA was from Biowest. Goat (GPMG-80A) and sheep (PAHPG-S) anti-human plasminogen antibodies were from ICL Lab and Haematologic Technologies, respectively. Trypsin, α-chymotrypsin, mannan, Zymosan A, D-Ala-Leu-Lys-7-amido-4-methylcoumarin, Triton X-100, and cold water fish gelatin were from Sigma Aldrich. Calcein AM was from BD Biosciences. Amboceptor, the guinea pig anti-sheep erythrocyte antibody, was of in-house origin from Aurora Hospital in Helsinki. Rat anti-CD59 antibody (YHT53.1) had been earlier produced in-house with a cell line originally kindly provided by Professor H. Waldmann (University of Oxford, England). HUVECs (passage 2) were the most generous gift from Mikko Helenius (University of Helsinki, Finland) and had been extracted from healthy human subjects as previously described [38]. EndoGRO-LS cell culture medium was from Merck Millipore. The RED-NHS kit for labeling proteins with NT-647 was from NanoTemper Technologies GmbH. Rabbit anti-C5b-9 antibody (A227) and human Factor H were from Complement Technology. PE/Cy5-labeled anti-P-selectin antibody (304908) was from BioLegend. Hirudin blood tubes were from Roche.

### Preparation of proteins

Plasminogen was extracted from citrated human plasma with Lysine HyperD affinity matrix (PALL Life Sciences) according to manufacturer’s instructions, followed by gel filtration (HiLoad^®^ 16/60 Superdex^®^ 200 pg, GE Healthcare) into 20 mM Hepes, pH 7.4, 144 mM NaCl, 2.5 mM MgCl_2_.

C3 and FH19-20 were prepared mainly as previously described [39, 40].

IgG was purified from human plasma with Protein G Sepharose 4 Fast Flow (Amersham Pharmacia Biotech AB) mainly according to the manufacturer’s instructions, followed by gel filtration into PBS. Heat aggregated immunoglobulin (HAIgG) was prepared by incubating this IgG for 20 min at 63°C.

To produce plasmin, plasminogen (1 mg/ml) was mixed with urokinase (16 μg/ml) in Hepes buffer on ice, transferred to 37°C for 5.5 min and then frozen on dry ice. The concentration of active plasmin was estimated to be 70% by comparing to the ability of a commercial product to cause a change in absorbance for the plasmin substrate S-2251 at 405 nm.

### Microtiter plate assay with fibrin coating

Microtiter plate wells (Nunc Maxisorp) were coated with 100μl of HAIgG (30 μg/ml), mannan (200 μg/ml), fibrinogen (100 μg/ml), or BSA (20 μg/ml) in PBS at 22°C for at least 3 hours. Fibrinogen-coated wells were treated with thrombin (100 μl, 5 units/ml) for 1 hour at 37°C. Wells were blocked with 0.5% BSA in PBS overnight at 4°C. After multiple rinses with PBS, wells were incubated with 2.5% serum diluted in Hepes buffer (20 mM Hepes, pH 7.4, 144 mM NaCl, 2.5 mM MgCl_2_, 5 mM CaCl_2_) for 30 min at 37°C. Formation of C3a was analyzed using the MicroVue C3a Plus EIA kit (Quidel).

### Preparation of fibrin beads

3 ml of fibrinogen (10 mg/ml) and 1 ml of thrombin (35 units/ml) in Hepes buffer were mixed and fibrin formation allowed for 1 hour at 37°C. The clot was frozen on dry ice, lyophilized for 44 hours and finally ground into very fine powder.

### Plasminogen activation on fibrin beads

Fibrin beads or human erythrocyte ghosts (both 120 μl/ml) in Hepes buffer with 1 mg/ml BSA were mixed with 3 μl of plasminogen (3 mg/ml) and incubated at 22°C for 40–60 min. Samples were washed with 1 ml of buffer. 400 μl of buffer was added, followed by 20 μl of plasmin substrate (D-Ala-Leu-Lys-7-amido-4-methylcoumarin, 1 mM) and 20 μl of tPA (20 units/ml). As a positive control, 3 μl of plasmin (8 μM) was used. After 30–45 min incubation at 22°C, the fluorescence of 100 μl-sample mixtures were measured with ex/em values 355/460nm (FLUOstar OPTIMA, BMG Labtech).

### Preparation of partially plasminogen-depleted serum

Goat anti-plasminogen antibody was coupled to CNBr-activated beads (GE Healthcare) according to manufacturer's instructions. The beads were washed into PBS, mixed with serum, and incubated on ice for 30 min with occasional stirring. The beads were centrifuged and the supernatant serum collected. By comparing the absorbances (at 280 nm), the depletion was estimated to dilute the serum by 40%. The amount of plasminogen removed from the partially depleted serum was analyzed with a dot blot using a sheep anti-plasminogen antibody and found to be 70, 70 and 50% in the different batches prepared.

### Complement activation in presence of fibrin beads

Activated Zymosan (0.2 mg) and fibrin beads (0.5 mg) were washed into Hepes buffer containing 1 mg/ml BSA. The beads were mixed with normal or partially plasminogen-depleted serum (final concentration 44% and volume 80 μl) and incubated at 37°C for 30 min. The sera were analyzed for complement activation as follows: (1) Formation of C3a was measured using the MicroVue C3a Plus EIA kit (Quidel). (2) Residual hemolytic activity was determined by incubating Es (1.5%) with factor H fragment comprising domains 19 and 20 (FH19-20; 60 μg/ml) and serum (4%) at 37°C for 25 min, and measuring absorbance of the supernatants at 414 nm.

### Hemolysis inhibition by plasmin

Hemolysis assays were done essentially as previously described [21]. 1% sheep erythrocytes (Es) were washed into 0.9% NaCl and mixed 1:1 with amboceptor (diluted 1:100 in 0.9% NaCl). Sensitized Es were washed into Hepes buffer. Potential inhibitor, human serum and Es were mixed in tubes on ice and then incubated at 37°C for 22 min. Samples were centrifuged for 3 min with 800 *g*. Absorbances of supernatants were measured at 414 nm. Within each sample, the erythrocyte and serum contents (volume-%) were kept equal, but they changed between the samples being 1.3, 1.5, 2.5, or 5.0%.

### Experiments with endothelial cells

HUVECs were cultured in serum-containing medium until passage 5 or 6. Cells from nearly confluent plates were detached and washed into PBS containing or not 0.1% gelatin. In the plasminogen binding assay, 3*10^5^ cells were incubated with NT-647-labeled plasminogen (0.3 mg/ml) in 100 μl PBS at 37°C for 30 min. Plasminogen binding was determined using CyAn^™^ ADP Analyzer.

For the complement activation assay, a fluorescent compound was introduced into the HUVECs by incubating 2*10^6^ cells in 600 μl gelatin PBS containing 10 μg Calcein AM at 37°C for 30 min, followed by two washes with gelatin PBS. In the cell lysis assay, samples of 4*10^4^ cells in a final volume of 80 μl were used. Cells were added with plasminogen (0.3 mg/ml) or the controls factor H (0.2 mg/ml) or BSA (0.3 mg/ml). Serum was added to a final concentration of 30%. As serum from healty human subjects does not lead to complement activation on endothelial cells [41] the cascade was initiated with anti-CD59 antibody, which activates the complement system through the classical pathway in addition to interfering with normal function of the complement regulator CD59. To the total lysis control sample, 3 μl of 9% Triton X-100 was added to force complete lysis. HUVECs were incubated at 37°C for 12 min. Cells were centrifuged and relative fluorescence of each supernatant measured at ex/em values 485/520nm (FLUOstar OPTIMA, BMG Labtech).

### Platelet isolation

Blood was collected into citrate tubes (Vacuette, 9 ml, Greiner Bio-One), followed by centrifugation at 200 *g* for 20 min at 22°C (no brake). Platelet rich plasma was mixed with citrate buffer (9.4 mM citrate, 4.8 mM citric acid, 17.4 mM dextrose, 145 mM NaCl, pH 6.5). Citric acid (0.25 M) was added to lower the pH of the sample to about 6.5 to prevent platelet activation during washes. Platelets were centrifuged at 440 *g* for 20 min, followed by two further washes and a final resuspension into modified Tyrode's buffer (137 mM NaCl, 2.7 mM KCl, 984 mM MgCl_2_, 7 mM Hepes, 0.35% BSA, 5.5 mM dextrose, 2 mM CaCl_2_, pH 7.4). Platelet concentration was determined with Sysmex KX-21 Hematology analyzer.

### Plasminogen binding to platelets

Platelets (4*10^6^ cells) were incubated with or without NT-647-labeled plasminogen (0.3 mg/ml) for 30 min at 22°C. Platelets were diluted in PBS and plasminogen binding determined immediately using the CyAn^™^ ADP Analyzer. Data was analyzed with the FlowJo-software.

### C3-fragment deposition on platelets

Platelets (4*10^6^ cells) were preincubated or not with plasminogen (0.2 mg/ml which equals the plasma concentration) at 22°C for 15 min. FH19-20 (0.2 mg/ml) was added to all samples to activate complement. In the negative control, activity was prevented using 10 mM EDTA. NT-647-labeled C3 (0.4 mg/ml) was added to all samples, followed by 30 μl of serum and a 15 min incubation at 37°C. Complement propagation was stopped with 10 mM EDTA and cells diluted in 2–4 ml PBS, followed by immediate flow cytometry analysis with the CyAn^™^ ADP Analyzer. Geometric mean fluorescence values were extracted from the flow cytometry data with the FlowJo-software.

### Platelet activation

Platelets (4*10^6^ cells) were preincubated with plasminogen (0.2 mg/ml), hirudin (10 μg/ml), hirudin (10 μg/ml) and FH19-20 (0.1 mg/ml), or buffer at 22°C for 15 min. Serum (or buffer) was added to a final concentration of 30% and platelets incubated at 37°C for 15 min. EDTA was added (10 mM final concentration), followed by 6 μl of anti-P-selectin antibody. Samples were incubated at 4°C for about 1 hour, and then analyzed by flow cytometry.

### Platelet aggregation

Platelets (6*10^6^ cells) in 30 μl of modified Tyrode's buffer were pipetted into wells of a 96-well microtiter plate. Plasminogen (2 μM) or buffer was added, and platelets were incubated at 22°C for at least 10 min. Alternatively, platelets were preincubated with FH19-20 (0.2 mg/ml). Serum mixed with hirudin (10 μg/ml) or buffer was added to the platelets to a final concentration of 30%. The platelets were agitated at 37°C in a microplate reader (FluoStar OPTIMA, BMG Labtech) with double orbital shaking mode. Absorbances at 405 nm were read every 19 seconds throughout the 20 min experiment.

### Statistical Methods

GraphPad Prism software was used to assess the statistical significance of observed differences. Both one-way ANOVA with Tukey's multiple comparison post-test and one-sample t-test were used.

## Results

### Complement system does not activate on fibrin surfaces

As fibrin has been claimed to activate the complement system [36, 37] and as it is a major site of plasminogen binding and activation *in vivo*, we considered it a possible model with which the effect of plasminogen on complement activation on a surface could be studied. Here, and in other assays of complement activation, we used serum and purified plasminogen. As serum contains plasminogen activators—albeit at lower levels than plasma—transformation of added plasminogen into plasmin on surfaces capable of plasminogen binding and activation was plausible. Further, some interfering reaction products might have been introduced into serum due to strong activation of coagulation during serum preparation.

**Fig 1:**
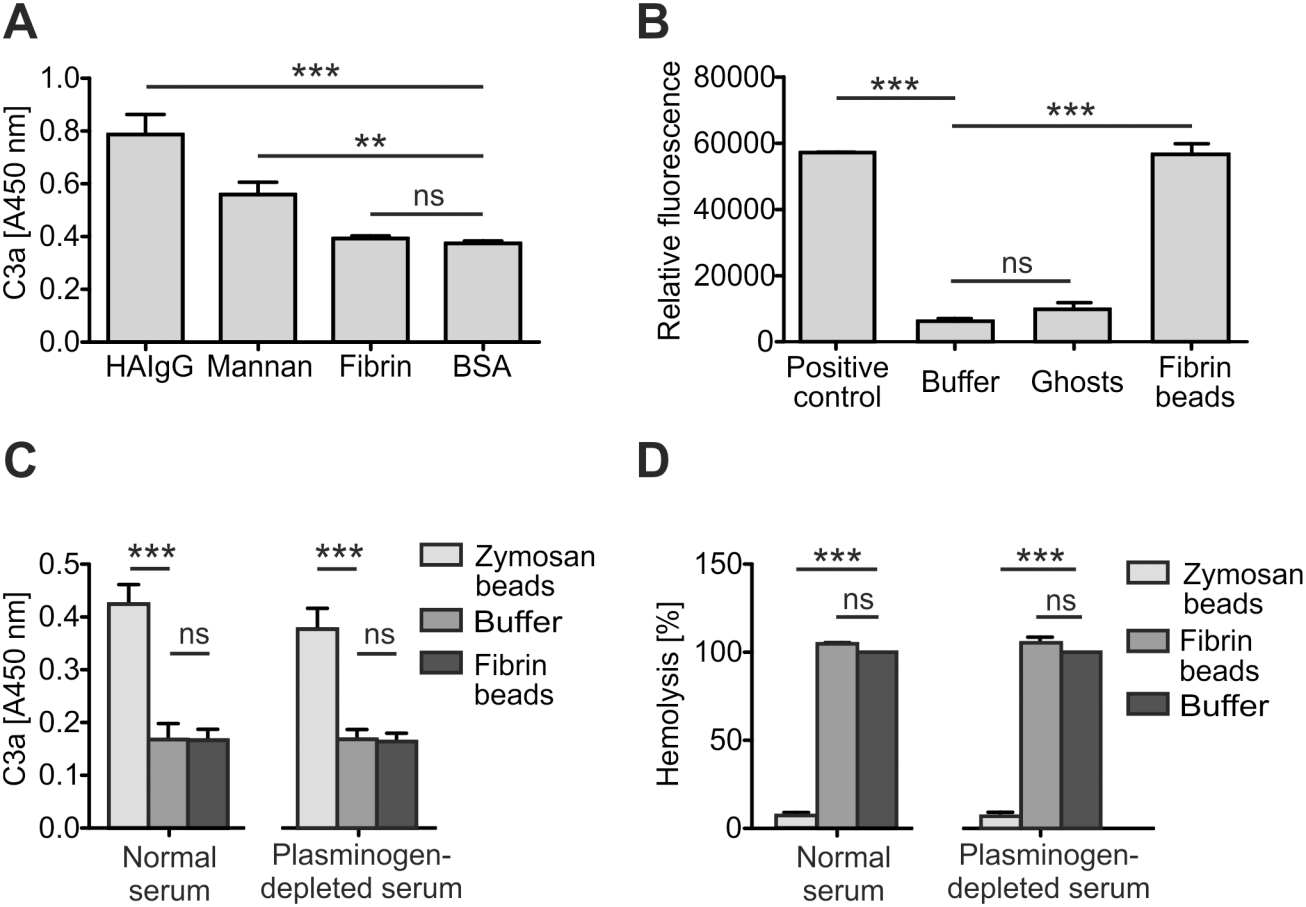
Complement system does not activate on fibrin surfaces. **A**: Formation of C3a in 2.5% serum incubated in wells coated with fibrin, the known complement activators heat aggregated immunoglobulin (HAIgG) and mannan, and the negative control BSA. The assay was done three times with triplicate measurements. **B**: Conversion of plasminogen to plasmin by tissue-type plasminogen activator (tPA) on fibrin beads. Formation of plasmin was followed by measuring appearance of a fluorescent cleavage product. Preformed plasmin was used as a positive and human erythrocyte ghosts as a negative control. The assay was done four times with single samples. **C**: Formation of C3a in normal and partially plasminogen-depleted serum incubated in the presence of fibrin beads. Buffer added or not with Zymosan beads served as controls. Three independent assays were analyzed in duplicate. **D**: Residual hemolytic activity of normal and partially plasminogen-depleted serum incubated in the presence of fibrin beads. Buffer added or not with Zymosan beads served as controls. Three independent assays were analyzed in triplicate. (Data shown are mean values ± SD. **, P < 0.01; ***, P < 0.001; ns, not significant).

First we coated fibrinogen on a microtiter plate and treated it with thrombin to make it more fibrin-like. When these wells were incubated with diluted serum, no difference in concentration of the complement activation product C3a could be observed compared to the negative control BSA (Fig 1A). With the positive controls, i.e. the known complement activators HAIgG and mannan, levels of C3a were increased (Fig 1A).

*In vivo*, fibrin appears as 3-D fibers, and we asked whether the lack of complement activation in our plate assay was due to an erroneous model. We next produced fibrin in the form of beads. These were considered a more suitable model of fibrin, since plasminogen bound to them could be further activated by tPA to plasmin as verified by cleavage of a plasmin substrate (Fig 1B). Ghosts of human erythrocytes, which practically do not bind plasminogen [33], were used as a negative control and expectedly showed no plasmin activity in the assay (Fig 1B).

Next it was reasoned that in the case plasminogen regulated complement on fibrin, then in normal serum complement activation would not occur on our beads. We therefore prepared serum that was mostly depleted of plasminogen. When fibrin beads were incubated with normal or the partially depleted serum, no difference in C3a-levels compared to the negative control could be observed (Fig 1C). The hemolytic activities were not affected either (Fig 1D). In contrast to this, incubation of normal and the partially depleted sera with the known complement activator Zymosan resulted in both increased production of C3a (Fig 1C) and loss of hemolytic activity (Fig 1D).

Taken together, the inability of fibrin to induce complement activation prevented us from using it as a model surface.

### Plasmin does not inhibit complement activation on erythrocytes at physiological concentrations

**Fig 2:**
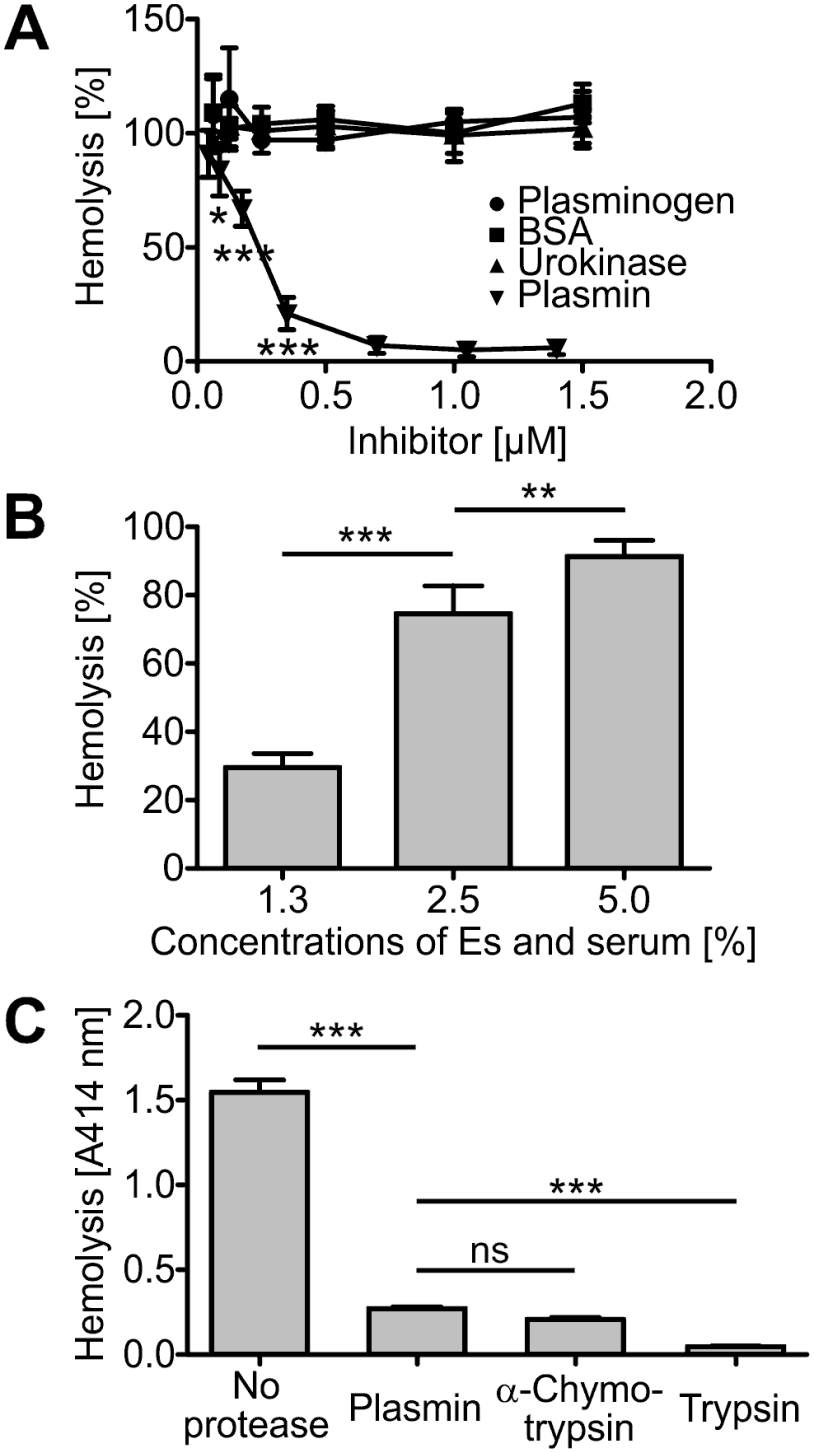
Plasminogen inhibits complement-mediated hemolysis only when artificially activated and present at disproportionately high concentration. **A**: Inhibition of erythrocyte lysis in highly diluted (1.5%) serum with increasing concentrations of added plasmin. Data for plasminogen, urokinase (used in preparation of plasmin), and the negative control BSA are also shown. The assay was done six times. **B**: Loss of plasmin-mediated inhibition of sheep erythrocytes (Es) with increasing concentration of serum. The concentration of plasmin was kept at 20% of the physiological plasma level. The assay was done six times. **C**: In addition to plasmin, hemolysis was inhibited with the proteases α-chymotrypsin and trypsin. The assay was done three times in triplicate. (Data shown are mean values ± SD. *, P < 0.05; **, P < 0.01; ***, P < 0.001; ns, not significant).

Of the cell types damaged in aHUS, erythrocytes have earlier been shown to be protected from complement-mediated hemolysis by plasmin in highly diluted serum [21]. Also in our assay plasmin (but not plasminogen) was observed to inhibit lysis of sensitized sheep erythrocytes (Fig 2A). But even though major inhibition of lysis was attained with lower than or close to physiological levels of plasminogen in both the reported [21] and our own assays, the amount of plasmin added was not in proportion to the concentrations of erythrocytes and serum used. For example, in the original assay described in the literature [21], 2 μM plasmin (as is found on average in 100% plasma) was added to 0.8% serum, resulting in a 100-fold excess concentration of plasmin compared to the serum used. Therefore we next fixed the amount of plasmin to a constant value of 0.4 μM (20% of normal plasma level) and tested inhibition with increasing concentrations of both erythrocytes and serum. Whereas most of hemolysis was prevented when only 1.5% cells and serum were present, the inhibition was nearly completely lost in mixtures with a serum concentration of only 5% (Fig 2B). These results show that plasmin prevents complement-mediated lysis only if the concentration of plasmin added exceeds multiple times the level of natural plasminogen in serum.

We also tested two unrelated serine proteases, the digestive system enzymes α-chymotrypsin and trypsin, for their ability to affect hemolysis. Similar to plasmin, both α-chymotrypsin and trypsin inhibited erythrocyte lysis at 1 μM concentrations (Fig 2C).

Taken together, these results confirm that plasmin (but not plasminogen) can inhibit hemolysis, but suggest that this occurs only if plasmin is exogenously activated and added in disproportionately high concentrations.

### Plasminogen does not inhibit complement activation on endothelial cells

**Fig 3:**
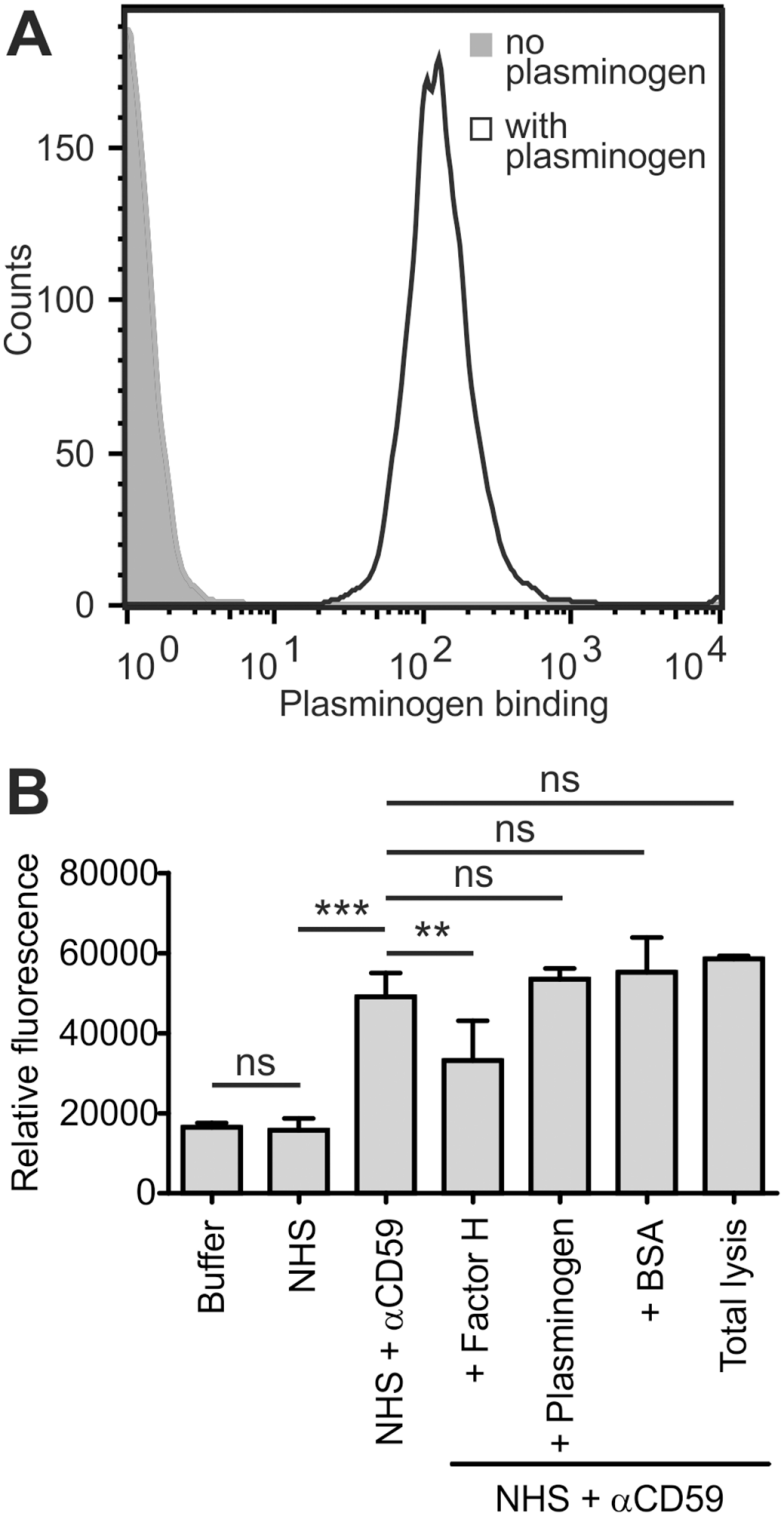
Plasminogen does not inhibit complement activation on endothelial cells. **A**: Confirmation of plasminogen binding to endothelial cells using flow cytometry. **B**: Effect of plasminogen on complement activation on human umbilical vein endothelial cells (HUVECs). Cells were incubated or not with plasminogen or the controls factor H or BSA prior to their exposure to 30% serum, followed by complement system activation using an anti-CD59 antibody. Complement activation was determined by measuring the release of a fluorescent compound introduced earlier into the cells. Representative data of an assay done five times are presented. (Shown are average values ± SD. **, P < 0.01; ***, P < 0.001; ns, not significant).

Damage to endothelial cells in aHUS is thought to involve poor complement regulation on them [42]. Unlike erythrocytes, endothelial cells are capable of not only binding but also of activating plasminogen to plasmin on their surfaces [2]. We therefore asked if complement activation could be regulated on endothelial cells by plasminogen. HUVECs were chosen as model cells for our assays, and first, efficient binding of fluorescently labeled plasminogen to these cells was confirmed with flow cytometry (Fig 3A). To study the effect of plasminogen on complement activation on endothelial cells, a fluorescent compound was introduced into HUVECs and its release during serum incubation used as a measure of complement cascade propagation. When the effect of plasminogen on cell lysis was experimented, no inhibition could be observed (Fig 3B), even though plasminogen was used in five times excess compared to the serum added. The positive control factor H, on the other hand, efficiently inhibited HUVEC lysis at levels similar to that of the 30% serum. These results indicate that plasminogen does not inhibit complement activation on resting endothelial cells.

### Plasminogen has a minor inhibitory effect on complement activation on platelets

**Fig 4:**
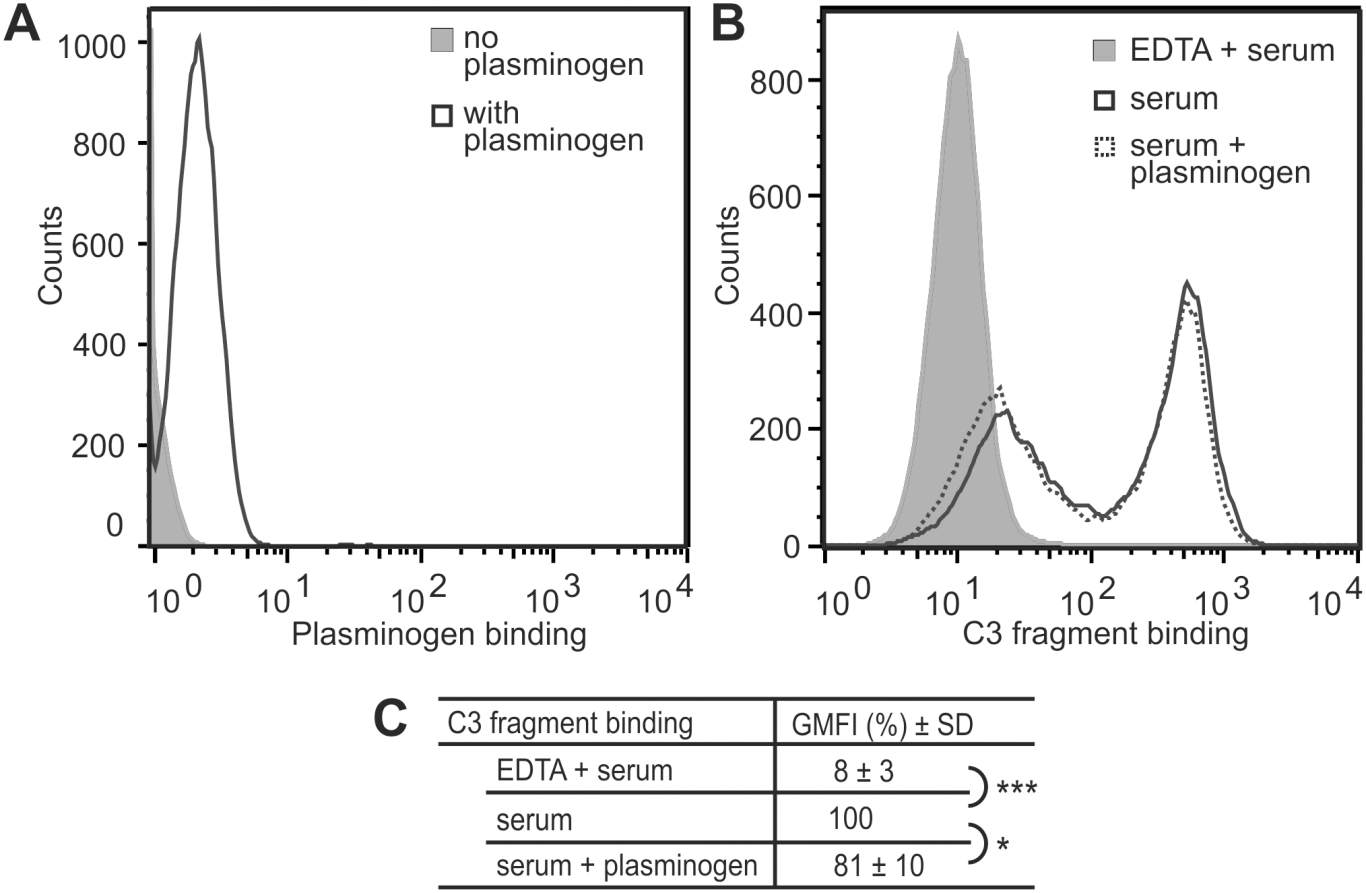
Plasminogen has a very weak inhibitory effect on complement deposition on platelets. **A**: Confirmation of plasminogen binding to platelets using flow cytometry. Shown is a representative histogram of four. **B**: A representative histogram showing very slight decrease in C3-fragment deposition on platelets in serum due to addition of plasminogen. Complement system activation was enabled with FH19-20. Representative histograms of an assay done five times are shown. **C**: Geometric mean fluorescence intensities of the assay described in (B). Values were compared to those for platelets incubated in the presence of serum only (100%). Shown are average values ± SD. *, P < 0.05.

The third group of cells affected in aHUS, i.e. platelets, have an exceptionally high capacity for plasminogen binding [33]. We used washed platelets in our assays, and first binding of fluorescently labeled plasminogen to these cells was confirmed (Fig 4A). To activate the complement system against platelets, we chose to use the C-terminal fragment of the complement regulator factor H [43]. This was expected not to interfere with putative regulative properties of plasminogen, since factor H and plasminogen interact with C3b noncompetitively through separate binding sites [21]. Addition of FH19-20 to serum expectedly resulted in increased deposition of C3 fragments on platelets (Fig 4B and 4C). When platelets were preincubated with plasminogen, a slight inhibitory effect on C3 deposition was observed (Fig 4B and 4C).

**Fig 5:**
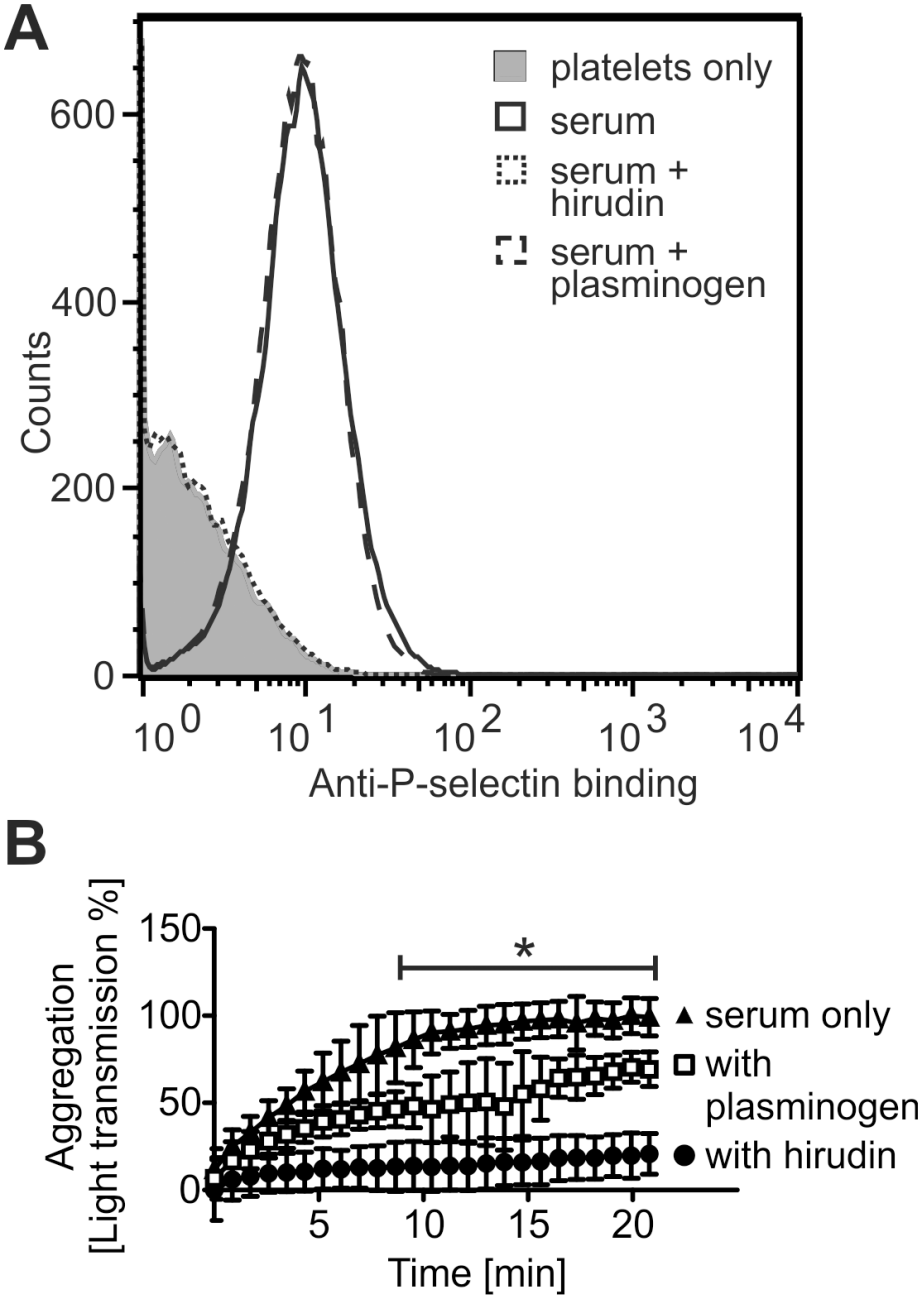
Plasminogen inhibits platelet aggregation but not their activation in serum. **A**: The activating effect of serum on platelets is counteracted with hirudin but not with plasminogen. Platelet activation was determined by detecting exposure of the activation marker P-selectin of platelet surfaces using flow cytometry. Shown are representative histograms of an assay performed three times. **B**: Inhibition of platelet aggregation in serum by addition of plasminogen and hirudin. Complete aggregation was set to 100% light transmission. Shown are mean values ± SD of an assay performed three times. For the time points marked with the line segment, absorbance values for samples incubated or not with plasminogen were compared (*, P < 0.05).

In line with an earlier study [44], also in our assays exposure of platelets to sera was observed to cause them to both get activated (Fig 5A) and aggregate (Fig 5B). As clotting blood involves formation of thrombin, we suspected some of this potent platelet agonist to carry over into the final product in preparation of serum. When hirudin was included in incubations, both activation (Fig 5A) and aggregation (Fig 5B) of platelets in serum were prevented, showing that thrombin was indeed responsible for the observed platelet-activating effect. When platelets were incubated with plasminogen prior to exposure to serum, activation of platelets was not affected (Fig 5A) but aggregation was markedly inhibited (Fig 5B).

**Fig 6:**
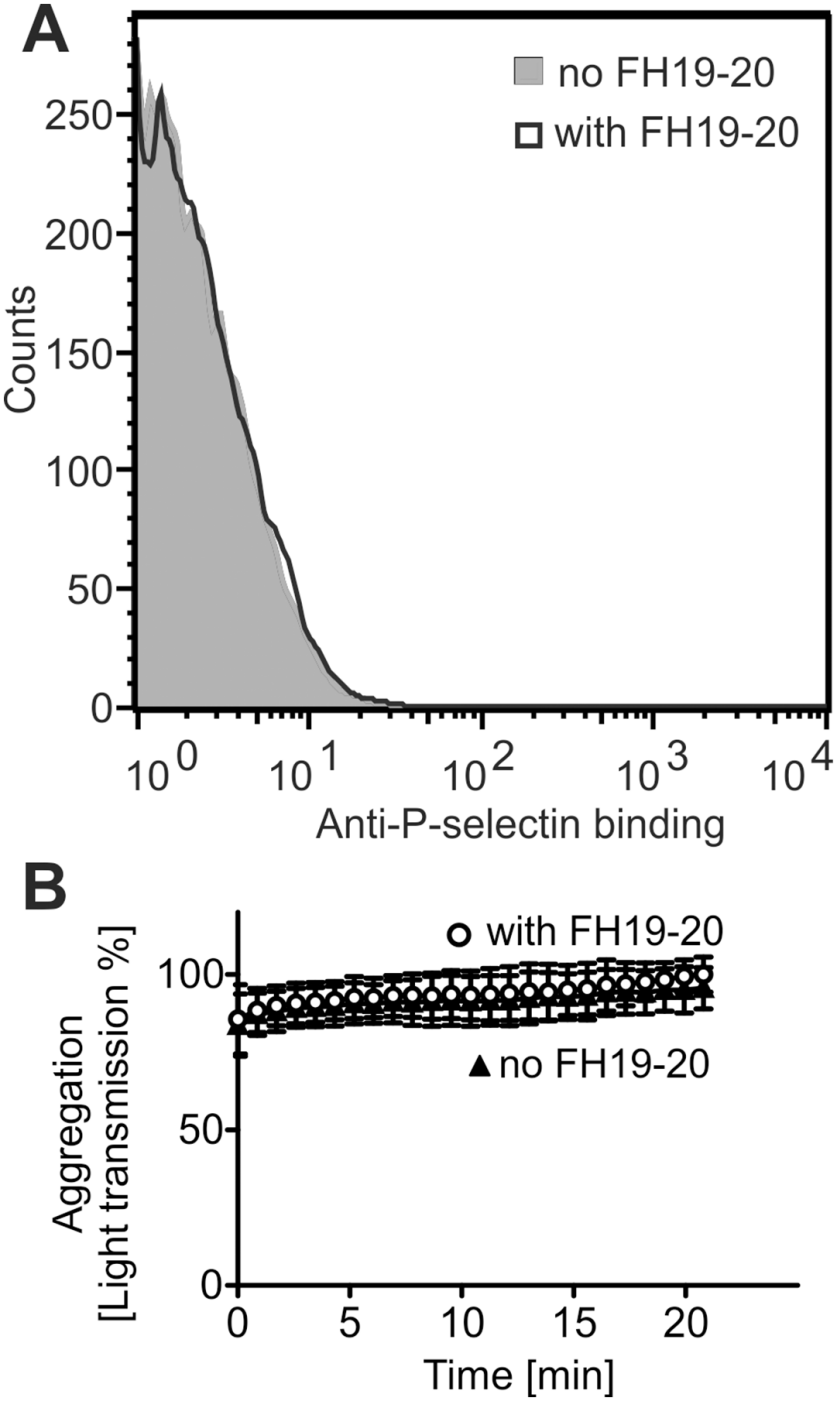
Complement activation alone does not cause platelet activation or aggregation. Platelets were incubated in serum and the complement system activated using FH19-20. Hirudin was included in the assays to prevent thrombin-mediated activation. **A**: Flow cytometry analysis of exposure of the activation marker P-selectin showed no activation of platelets due to complement activation. Shown are representative histograms of an assay performed three times. **B**: Complement activation did not lead to platelet aggregation. Complete aggregation was set to 100% light transmission. Shown are average values of an assay performed three times.

It has previously been shown that thrombin-mediated platelet activation and aggregation are enhanced in presence of complement [45], but purified C3a alone cannot induce aggregation [46]. In line with this, in our assays, complement activation as such (in the absence of specific platelet agonists) was not able to activate or aggregate platelets (Fig 6A and 6B).

Taken together, our results show that plasminogen has a very modest inhibitory effect on complement deposition on platelets, but a prominent restrictive effect on aggregation.

## Discussion

aHUS features lysis of red blood cells, swelling and detachment of endothelial cells especially in the glomeruli, and low platelet count due to their activation and consumption in thrombus formation in microvasculature [10]. Whereas loss of proper control over complement activation is long established to be central to aHUS [11], recent years have witnessed the emergence of some coagulation and fibrinolytic proteins as mediators of the disease [9, 16, 17]. In this work we studied the regulatory properties of the key fibrinolytic protein plasminogen on complement activation on surfaces relevant to aHUS. Our results show that plasminogen has a very slight inhibitory effect on complement activation on platelets but not on erythrocytes or endothelial cells.

Fibrin is not considered a target of complement activation in aHUS, but it was included in this study for its ability to bind and activate plasminogen. The discrepancy between earlier work claiming complement activation on fibrin and our contradictory result can, at least partially, be explained by experimental differences. For example, Endo *et al*. [37] used mouse serum and untreated fibrinogen to show a modest increase above background in C4-deposition. Shats-Tseytlina *et al*. [36] observed an increase in production of C3a in the presence of a fibrin clot compared to buffer alone, but no positive control was provided to judge the practical relevance of this. In any case, an artificial fibrin surface created in a buffer is presumably not representative of a clot *in vivo*. It lacks all the regulatory factors like α2-antiplasmin immobilized on natural fibrin networks [5], which are likely to affect the function of at least plasminogen on the fibers.

Of the cells affected in aHUS, erythrocytes have previously been shown to be protected from complement-mediated lysis by the protease plasmin in highly diluted serum *in vitro* [21]. We were able to confirm this (Fig 2A), but when serum concentration was increased, the protective effect of plasmin was lost (Fig 2B). This was presumable mostly due to the need to overcome the effect of the natural plasmin inhibitors of plasma. As erythrocytes are incapable of binding plasminogen [33], they cannot provide protection from plasmin inhibitors present in plasma and free plasmin is quickly inactivated by α2-antiplasmin [4]. Further, plasmin can deactivate complement proteins in plasma [22], and it most likely functioned also in our serum assay by digesting complement components. The central complement members C4 and C3 are rather abundant proteins in plasma [47, 48], and increasing the serum concentration probably overwhelmed the proteolytic capacity of plasmin added. Taken together, (i) plasminogen is not activated to plasmin on erythrocytes themselves, (ii) the inhibitors of plasma prevent diffusion of active plasmin onto erythrocytes from other locations, and (iii) the abundance of target complement proteins to be digested by plasmin necessitates many times more plasmin that can theoretically form of the 2 μM plasminogen that is naturally present. We seriously doubt that *in vivo* a high enough local concentration of free plasmin could be attained on the surface of an erythrocyte to allow significant effect on complement activation.

Endothelial cells, the other important group of cells severely damaged in aHUS, are capable of not only binding but also of activating plasminogen on their surfaces [2]. Considering the reported regulative properties of both plasminogen and plasmin on complement proteins mainly in fluid phase [21–25, 27, 28], inhibition of complement propagation on endothelial cell surfaces by plasminogen was seen highly plausible. Somewhat disappointingly, however, no inhibition of HUVEC lysis in serum by adding plasminogen was observed (Fig 3B). This can possibly be attributed to the very weak enhancing effect of the zymogen plasminogen on C3b inactivation [21]. As we did not specifically activate the HUVECs and as only 30% serum containing quite little tPA was used, not much conversion of plasminogen to the more effective protease form plasmin is expected to have occurred. It is possible that on activated endothelial cells and in the presence of higher serum levels a restrictive effect for plasminogen would be observed. We did not, however, proceed to experiment the matter further. This was because only a minor inhibitory effect for plasminogen on complement activation on platelets was observed (Fig 4B), even though platelets have an exceptionally high plasminogen binding capacity [33] and the activated state of platelets (Fig 5A) enhances plasmin formation on their surfaces [49].

Similar to endothelial cells, platelets of aHUS-patients with mutations in the complement regulator FH show excessive deposition of complement fragments on their surfaces [34]. Analysis of patient platelets has revealed the presence of markers of cell activation on them as well [34]. These two processes, i.e. activation of the complement system and of platelets, seem to be reciprocally intertwined [50]. On one hand, complement enhances thrombin- and ADP-mediated platelet activation [45], whereas in the absence of C3, a modest attenuation of aggregation is observed for platelets activated with an agonist peptide [51]. On the other hand, activation of platelets can lead to activation of the complement system. For example, increased expression of the receptor for C1q [52] enables triggering of the classical pathway of complement [53], whereas exposure of P-selectin initiates the alternative pathway through binding C3b [50], albeit weakly. All this suggests that by inhibiting complement activation, formation of thrombi could be hindered at least to some extent.

In our experiments incubation of platelets with plasminogen, prior to their exposure to serum, inhibited deposition of C3 fragments on cells, but the effect was very weak (Fig 4). In line with the earlier reported failure of C3a alone to bring about platelet aggregation [46], directing complement attack against platelets in the absence of specific agonists (like thrombin) was not enough to cause them to activate or aggregate (Fig 6A and 6B). This suggests only a modifying role for complement on these processes. Considering that even in the case of complete absence of complement activation only a modest attenuation of platelet aggregation has been reported [51], the observed minor restriction of complement propagation by plasminogen is not expected to be significant *in vivo*.

But why is plasminogen deficiency then associated with the thrombotic desease aHUS if not for impaired complement regulation? Thrombi are composed of a mesh of fibrin fibers and aggregated platelets. Besides causing inefficient fibrinolysis [9], plasminogen deficiency could be involved in increased platelet aggregation. Interestingly, we observed a clear inhibitory effect of plasminogen on platelet aggregation in serum (Fig 5B). As plasminogen was unable to prevent the thrombin-mediated activation of platelets (Fig 5A), it must have exerted its effect further downstream. Earlier it has been shown that the protease plasmin is capable of disintegrating existing platelet aggregates [54]. We used purified plasminogen in our assays, but formation of plasmin was nevertheless likely. This was because the serum contained thrombin (Fig 5) which activates platelets and increases plasminogen binding to them multifold [55], and because platelet activation markedly enhances conversion of plasminogen to plasmin by tPA [49]. Platelet disaggregation due to fibrinogenolysis [54], on the other hand, was unlikely since we used only serum with very low fibrinogen content. Instead, plasmin might have cleaved platelet surface receptors important for adhesion. Supporting this, plasmin has been shown to severely impair the functional integrity of, for example, platelet surface glycoproteins Ib and IIb/IIIa [56]. All in all, we propose that impaired control over thrombus growth either through diminished fibrinolysis [9] or enhanced platelet aggregation explains the newly described link between lack of plasminogen and aHUS.

But since plasminogen deficiency alone does not cause thrombosis [8], additional abnormalities are needed for the thrombotic events to manifest in plasminogen deficient aHUS-patients. Albeit not due to impaired regulation by plasminogen, malfunction of the complement system could certainly be involved. Namely, the three aHUS-patients with variation in plasminogen encoding gene were reported to carry one or two additional nucleotide changes in the complement genes *C1S*, *C3*, *C8A*, *CFHR5*, and *C9* [9]. It is difficult, however, to vision how for example the reported gene variation causing C9 deficiency [9] could lead to excessive complement activation. Importantly, most recent research on DGKε-associated aHUS [57] forces us to acknowledge that the disease might evolve through mechanisms unrelated to the complement system. Loss of function of the kinase DGKε leads to decreased survival and a prothrombotic phenotype of at least endothelial cells rather than to complement-mediated lesions [57]. DGKε is also found in platelets [15] where it functions to inhibit their activation [58]. Data on the response of the aHUS-patients with plasminogen deficiency to treatment with the therapeutic anti-C5 antibody eculizumab was not reported [9]. When available, it will help in answering the question of involvement of complement activation in the disease pathogenesis in these patients.

In conclusion, we have investigated the regulatory properties of plasminogen on complement activation on cell surfaces relevant to aHUS. The very weak inhibitory role of plasminogen, observed only on platelets, suggests that plasminogen is not important in complement regulation. Therefore the association of plasminogen deficiency with aHUS cannot be explained by poor control over complement propagation. Instead, plasminogen was shown to have a marked inhibitory effect on platelet aggregation in serum. We propose that, in aHUS patients with a reduced level of plasminogen, the lack of this zymogen leads to reduced proteolytic activity of plasmin and thereby growth of thrombi in the microvasculature. Besides impaired plasmin activity, the possibility of simultaneous malfunction of the complement system in these patients remains. The emergence of non-complement proteins as mediators of aHUS, however, prompts future research to broaden the scope outside of complement dysregulation.

## References

[1] Weisel JW, Litvinov RI. The biochemical and physical process of fibrinolysis and effects of clot structure and stability on the lysis rate. Cardiovascular & hematological agents in medicinal chemistry. 2008;6(3):161–80. Epub 2008/08/05. . 18673231 10.2174/187152508784871963

[2] Madureira PA, Surette AP, Phipps KD, Taboski MA, Miller VA, Waisman DM. The role of the annexin A2 heterotetramer in vascular fibrinolysis. Blood. 2011;118(18):4789–97. Epub 2011/09/13. 10.1182/blood-2011-06-334672 . 21908427

[3] Cesarman-Maus G, Hajjar KA. Molecular mechanisms of fibrinolysis. British journal of haematology. 2005;129(3):307–21. Epub 2005/04/22. 10.1111/j.1365-2141.2005.05444.x . 15842654

[4] Schaller J, Gerber SS. The plasmin-antiplasmin system: structural and functional aspects. Cellular and molecular life sciences: CMLS. 2011;68(5):785–801. Epub 2010/12/08. 10.1007/s00018-010-0566-5 . 21136135 PMC11115092

[5] Sakata Y, Aoki N. Cross-linking of alpha 2-plasmin inhibitor to fibrin by fibrin-stabilizing factor. The Journal of clinical investigation. 1980;65(2):290–7. ; PubMed Central PMCID: PMCPmc371366. 6444305 10.1172/JCI109671 PMC371366

[6] Deryugina EI, Quigley JP. Cell surface remodeling by plasmin: a new function for an old enzyme. Journal of biomedicine & biotechnology. 2012;2012:564259 Epub 2012/10/26. 10.1155/2012/564259 ; PubMed Central PMCID: PMCPmc3477900. 23097597 PMC3477900

[7] Drew AF, Kaufman AH, Kombrinck KW, Danton MJ, Daugherty CC, Degen JL, et al Ligneous conjunctivitis in plasminogen-deficient mice. Blood. 1998;91(5):1616–24. Epub 1998/03/21. . 9473227

[8] Mehta R, Shapiro AD. Plasminogen deficiency. Haemophilia. 2008;14(6):1261–8. 10.1111/j.1365-2516.2008.01825.x 19141167

[9] Bu F, Maga T, Meyer NC, Wang K, Thomas CP, Nester CM, et al Comprehensive genetic analysis of complement and coagulation genes in atypical hemolytic uremic syndrome. Journal of the American Society of Nephrology: JASN. 2014;25(1):55–64. Epub 2013/09/14. 10.1681/asn.2013050453 24029428 PMC3871781

[10] Noris M, Remuzzi G. Atypical hemolytic-uremic syndrome. The New England journal of medicine. 2009;361(17):1676–87. Epub 2009/10/23. 10.1056/NEJMra0902814 . 19846853

[11] Kavanagh D, Goodship T. Genetics and complement in atypical HUS. Pediatric nephrology (Berlin, Germany). 2010;25(12):2431–42. Epub 2010/06/08. 10.1007/s00467-010-1555-5 20526633 PMC2962786

[12] Ricklin D, Hajishengallis G, Yang K, Lambris JD. Complement: a key system for immune surveillance and homeostasis. Nature immunology. 2010;11(9):785–97. Epub 2010/08/20. 10.1038/ni.1923 20720586 PMC2924908

[13] Loirat C, Fremeaux-Bacchi V. Atypical hemolytic uremic syndrome. Orphanet journal of rare diseases. 2011;6:60 Epub 2011/09/10. 10.1186/1750-1172-6-60 21902819 PMC3198674

[14] Modde F, Agustian PA, Wittig J, Dammrich ME, Forstmeier V, Vester U, et al Comprehensive analysis of glomerular mRNA expression of pro- and antithrombotic genes in atypical haemolytic-uremic syndrome (aHUS). Virchows Archiv: an international journal of pathology. 2013;462(4):455–64. Epub 2013/03/12. 10.1007/s00428-013-1386-4 . 23475501

[15] Lemaire M, Fremeaux-Bacchi V, Schaefer F, Choi M, Tang WH, Le Quintrec M, et al Recessive mutations in DGKE cause atypical hemolytic-uremic syndrome. Nature genetics. 2013;45(5):531–6. Epub 2013/04/02. 10.1038/ng.2590 23542698 PMC3719402

[16] Delvaeye M, Noris M, De Vriese A, Esmon CT, Esmon NL, Ferrell G, et al Thrombomodulin mutations in atypical hemolytic-uremic syndrome. The New England journal of medicine. 2009;361(4):345–57. Epub 2009/07/25. 10.1056/NEJMoa0810739 19625716 PMC3530919

[17] Matsukuma E, Gotoh Y, Kuroyanagi Y, Yamada T, Iwasa M, Yamakawa S, et al A case of atypical hemolytic uremic syndrome due to anti-factor H antibody in a patient presenting with a factor XII deficiency identified two novel mutations. Clinical and experimental nephrology. 2011;15(2):269–74. Epub 2011/01/29. 10.1007/s10157-010-0375-z . 21271273

[18] Markiewski MM, Nilsson B, Ekdahl KN, Mollnes TE, Lambris JD. Complement and coagulation: strangers or partners in crime? Trends in immunology. 2007;28(4):184–92. Epub 2007/03/06. 10.1016/j.it.2007.02.006 . 17336159

[19] Oikonomopoulou K, Ricklin D, Ward PA, Lambris JD. Interactions between coagulation and complement—their role in inflammation. Seminars in immunopathology. 2012;34(1):151–65. Epub 2011/08/04. 10.1007/s00281-011-0280-x ; PubMed Central PMCID: PMCPmc3372068. 21811895 PMC3372068

[20] Ghebrehiwet B, Randazzo BP, Dunn JT, Silverberg M, Kaplan AP. Mechanisms of activation of the classical pathway of complement by Hageman factor fragment. The Journal of clinical investigation. 1983;71(5):1450–6. Epub 1983/05/01. 6304147 10.1172/JCI110898 PMC437009

[21] Barthel D, Schindler S, Zipfel PF. Plasminogen is a complement inhibitor. The Journal of biological chemistry. 2012;287(22):18831–42. Epub 2012/03/28. 10.1074/jbc.M111.323287 22451663 PMC3365705

[22] Pillemer L, Ratnoff OD, Blum L, Lepow IH. The inactivation of complement and its components by plasmin. The Journal of experimental medicine. 1953;97(4):573–89. Epub 1953/04/01. 13052820 10.1084/jem.97.4.573 PMC2136289

[23] Ward PA. A plasmin-split fragment of C'3 as a new chemotactic factor. The Journal of experimental medicine. 1967;126(2):189–206. Epub 1967/08/01. 4226271 10.1084/jem.126.2.189 PMC2138318

[24] Taubman SB, Lepow IH. Clevage of human Cls by human lysosomal enzymes, plasmin, and trypsin. Immunochemistry. 1971;8(10):951–61. Epub 1971/10/01. . 4261121 10.1016/0019-2791(71)90433-2

[25] Ikari N, Niinobe M, Fujii S. Proteolysis of factor B by plasma kallikrein and plasmin. FEBS letters. 1981;131(1):143–6. Epub 1981/08/17. . 6456937 10.1016/0014-5793(81)80906-4

[26] Seya T, Nagasawa S, Matsukura M, Hasegawa H, Atkinson JP. Generation of C3d,g and C3d by urokinase-treated plasma in association with fibrinolysis. Complement (Basel, Switzerland). 1985;2(2–3):165–74. Epub 1985/01/01. . 2935360 10.1159/000467857

[27] Amara U, Flierl MA, Rittirsch D, Klos A, Chen H, Acker B, et al Molecular intercommunication between the complement and coagulation systems. Journal of immunology (Baltimore, Md: 1950). 2010;185(9):5628–36. Epub 2010/09/28. 10.4049/jimmunol.0903678 20870944 PMC3123139

[28] Foley JH, Peterson EA, Lei V, Wan LW, Krisinger MJ, Conway EM. Interplay between fibrinolysis and complement: plasmin cleavage of iC3b modulates immune responses. Journal of thrombosis and haemostasis: JTH. 2015;13(4):610–8. Epub 2015/01/06. 10.1111/jth.12837 . 25556624

[29] Rooijakkers SH, van Wamel WJ, Ruyken M, van Kessel KP, van Strijp JA. Anti-opsonic properties of staphylokinase. Microbes and infection / Institut Pasteur. 2005;7(3):476–84. Epub 2005/03/29. 10.1016/j.micinf.2004.12.014 . 15792635

[30] Chung MC, Tonry JH, Narayanan A, Manes NP, Mackie RS, Gutting B, et al Bacillus anthracis interacts with plasmin(ogen) to evade C3b-dependent innate immunity. PloS one. 2011;6(3):e18119 Epub 2011/04/06. 10.1371/journal.pone.0018119 21464960 PMC3064659

[31] Vieira ML, de Morais ZM, Vasconcellos SA, Romero EC, Nascimento AL. In vitro evidence for immune evasion activity by human plasmin associated to pathogenic Leptospira interrogans. Microbial pathogenesis. 2011;51(5):360–5. Epub 2011/08/02. 10.1016/j.micpath.2011.06.008 . 21802507

[32] Lahteenmaki K, Kuusela P, Korhonen TK. Bacterial plasminogen activators and receptors. FEMS microbiology reviews. 2001;25(5):531–52. Epub 2001/12/18. . 11742690 10.1111/j.1574-6976.2001.tb00590.x

[33] Miles LA, Plow EF. Binding and activation of plasminogen on the platelet surface. The Journal of biological chemistry. 1985;260(7):4303–11. Epub 1985/04/10. . 3920216

[34] Stahl AL, Vaziri-Sani F, Heinen S, Kristoffersson AC, Gydell KH, Raafat R, et al Factor H dysfunction in patients with atypical hemolytic uremic syndrome contributes to complement deposition on platelets and their activation. Blood. 2008;111(11):5307–15. Epub 2008/02/13. 10.1182/blood-2007-08-106153 . 18268093

[35] Karnchanaphanurach P, Mirchev R, Ghiran I, Asara JM, Papahadjopoulos-Sternberg B, Nicholson-Weller A, et al C3b deposition on human erythrocytes induces the formation of a membrane skeleton-linked protein complex. The Journal of clinical investigation. 2009;119(4):788–801. Epub 2009/03/05. 10.1172/jci36088 19258706 PMC2662546

[36] Shats-Tseytlina EA, Nair CH, Dhall DP. Complement activation: a new participant in the modulation of fibrin gel characteristics and the progression of atherosclerosis? Blood coagulation & fibrinolysis: an international journal in haemostasis and thrombosis. 1994;5(4):529–35. Epub 1994/08/01. . 7841309

[37] Endo Y, Nakazawa N, Iwaki D, Takahashi M, Matsushita M, Fujita T. Interactions of ficolin and mannose-binding lectin with fibrinogen/fibrin augment the lectin complement pathway. Journal of innate immunity. 2010;2(1):33–42. Epub 2010/04/09. 10.1159/000227805 . 20375621

[38] Yegutkin GG, Henttinen T, Jalkanen S. Extracellular ATP formation on vascular endothelial cells is mediated by ecto-nucleotide kinase activities via phosphotransfer reactions. FASEB journal: official publication of the Federation of American Societies for Experimental Biology. 2001;15(1):251–60. Epub 2001/01/10. 10.1096/fj.00-0268com . 11149913

[39] Koistinen V, Wessberg S, Leikola J. Common binding region of complement factors B, H and CR1 on C3b revealed by monoclonal anti-C3d. Complement and inflammation. 1989;6(4):270–80. Epub 1989/01/01. . 2527715 10.1159/000463102

[40] Lehtinen MJ, Rops AL, Isenman DE, van der Vlag J, Jokiranta TS. Mutations of factor H impair regulation of surface-bound C3b by three mechanisms in atypical hemolytic uremic syndrome. The Journal of biological chemistry. 2009;284(23):15650–8. Epub 2009/04/09. 10.1074/jbc.M900814200 19351878 PMC2708861

[41] Noris M, Galbusera M, Gastoldi S, Macor P, Banterla F, Bresin E, et al Dynamics of complement activation in aHUS and how to monitor eculizumab therapy. Blood. 2014;124(11):1715–26. Epub 2014/07/20. 10.1182/blood-2014-02-558296 ; PubMed Central PMCID: PMCPmc4162105. 25037630 PMC4162105

[42] Manuelian T, Hellwage J, Meri S, Caprioli J, Noris M, Heinen S, et al Mutations in factor H reduce binding affinity to C3b and heparin and surface attachment to endothelial cells in hemolytic uremic syndrome. The Journal of clinical investigation. 2003;111(8):1181–90. Epub 2003/04/17. 10.1172/jci16651 12697737 PMC152934

[43] Saggu G, Cortes C, Emch HN, Ramirez G, Worth RG, Ferreira VP. Identification of a novel mode of complement activation on stimulated platelets mediated by properdin and C3(H2O). Journal of immunology (Baltimore, Md: 1950). 2013;190(12):6457–67. Epub 2013/05/17. 10.4049/jimmunol.1300610 23677468 PMC3784323

[44] Licht C, Pluthero FG, Li L, Christensen H, Habbig S, Hoppe B, et al Platelet-associated complement factor H in healthy persons and patients with atypical HUS. Blood. 2009;114(20):4538–45. Epub 2009/08/26. 10.1182/blood-2009-03-205096 . 19704120

[45] Polley MJ, Nachman R. The human complement system in thrombin-mediated platelet function. The Journal of experimental medicine. 1978;147(6):1713–26. Epub 1978/06/01. 681879 10.1084/jem.147.6.1713 PMC2184322

[46] Polley MJ, Nachman RL. Human platelet activation by C3a and C3a des-arg. The Journal of experimental medicine. 1983;158(2):603–15. Epub 1983/08/01. 6604123 10.1084/jem.158.2.603 PMC2187348

[47] Schreiber RD, Müller-Eberhard HJ. FOURTH COMPONENT OF HUMAN COMPLEMENT: DESCRIPTION OF A THREE POLYPEPTIDE CHAIN STRUCTURE. The Journal of experimental medicine. 1974;140(5):1324–35. ; PubMed Central PMCID: PMCPmc2139723. 4424566 10.1084/jem.140.5.1324 PMC2139723

[48] Müller-Eberhard HJ, Nilsson U, Aronsson T. ISOLATION AND CHARACTERIZATION OF TWO β(1)-GLYCOPROTEINS OF HUMAN SERUM. The Journal of experimental medicine. 1960;111(2):201–15. ; PubMed Central PMCID: PMCPmc2137253. 13726744 10.1084/jem.111.2.201 PMC2137253

[49] Loscalzo J, Pasche B, Ouimet H, Freedman JE. Platelets and plasminogen activation. Thrombosis and haemostasis. 1995;74(1):291–3. Epub 1995/07/01. . 8578474

[50] Del Conde I, Cruz MA, Zhang H, Lopez JA, Afshar-Kharghan V. Platelet activation leads to activation and propagation of the complement system. The Journal of experimental medicine. 2005;201(6):871–9. Epub 2005/03/23. 10.1084/jem.20041497 15781579 PMC2213112

[51] Gushiken FC, Han H, Li J, Rumbaut RE, Afshar-Kharghan V. Abnormal platelet function in C3-deficient mice. Journal of thrombosis and haemostasis: JTH. 2009;7(5):865–70. Epub 2009/03/18. 10.1111/j.1538-7836.2009.03334.x 19291167 PMC2867673

[52] Peerschke EI, Murphy TK, Ghebrehiwet B. Activation-dependent surface expression of gC1qR/p33 on human blood platelets. Thrombosis and haemostasis. 2003;89(2):331–9. Epub 2003/02/08. . 12574814

[53] Peerschke EI, Yin W, Grigg SE, Ghebrehiwet B. Blood platelets activate the classical pathway of human complement. Journal of thrombosis and haemostasis: JTH. 2006;4(9):2035–42. Epub 2006/09/12. 10.1111/j.1538-7836.2006.02065.x . 16961611

[54] Loscalzo J, Vaughan DE. Tissue plasminogen activator promotes platelet disaggregation in plasma. The Journal of clinical investigation. 1987;79(6):1749–55. Epub 1987/06/01. 10.1172/jci113015 2438305 PMC424517

[55] Miles LA, Ginsberg MH, White JG, Plow EF. Plasminogen interacts with human platelets through two distinct mechanisms. The Journal of clinical investigation. 1986;77(6):2001–9. Epub 1986/06/01. 10.1172/jci112529 3086385 PMC370561

[56] Hoffmann JJ, Janssen WC. Interactions between thrombolytic agents and platelets: effects of plasmin on platelet glycoproteins Ib and IIb/IIIa. Thrombosis research. 1992;67(6):711–9. Epub 1992/09/15. . 1440536 10.1016/0049-3848(92)90075-l

[57] Bruneau S, Neel M, Roumenina LT, Frimat M, Laurent L, Fremeaux-Bacchi V, et al Loss of DGKepsilon induces endothelial cell activation and death independently of complement activation. Blood. 2015;125(6):1038–46. Epub 2014/12/17. 10.1182/blood-2014-06-579953 . 25498910

[58] Nunn DL, Watson SP. A diacylglycerol kinase inhibitor, R59022, potentiates secretion by and aggregation of thrombin-stimulated human platelets. The Biochemical journal. 1987;243(3):809–13. Epub 1987/05/01. 2821994 10.1042/bj2430809 PMC1147929

